# P-2162. Utility of Metagenomic Sequencing of Microbial Plasma Cell-Free DNA with Different Testing Strategies

**DOI:** 10.1093/ofid/ofae631.2316

**Published:** 2025-01-29

**Authors:** Hutton Brandon, Katherine Arn, Brandon J Webb, Bert K Lopansri, Hannah Imlay

**Affiliations:** University of Utah Health, Salt Lake City, Utah; University of Utah Health, Salt Lake City, Utah; Intermountain Healthcare, Murray, UT; Intermountain Healthcare, Murray, UT; University of Utah Health, Salt Lake City, Utah

## Abstract

**Background:**

While promising, optimal use criteria for metagenomic next-generation sequencing of microbial cell-free DNA (mcfDNA) to diagnose infectious diseases (ID) are not yet established. We examined this question by comparing the real-world clinical impact of mcfDNA testing on ID management in two academic medical systems with similar patient case mix but differing test ordering practices. Both institutions used the Karius assay (Redwood City, CA). Intermountain Health (IH) used an expedited pathway for specimen shipping and restricted test ordering to ID physicians with suggested use criteria. By contrast, at University of Utah Health (UU), testing occurred without systemic education and lab processing was slower.

Demographics

**Figure:**
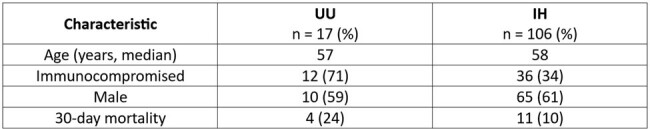

**Methods:**

We conducted a retrospective study of all mcfDNA testing at IH and UU from April 2022 – June 2023. We compared indications, incidence (per ID consult), and turn-around-time (TAT) for testing at each institution. Clinical impact of mcfDNA testing was evaluated for each institution using published criteria (Table 2).

Clinical Impact Criteria

**Figure:**
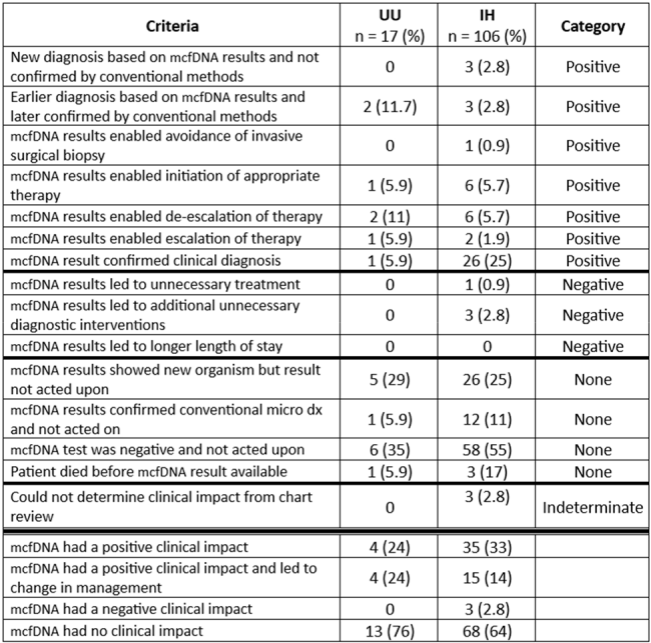

UU, University of Utah; IH, Intermountain Health; mcfDNA, metagenomic next generation sequencing of plasma microbial cell-free DNA.

Criteria adapted from Hogan CA, Yang S, Garner OB, et al. Clinical Impact of Metagenomic Next-Generation Sequencing of Plasma Cell-Free DNA for the Diagnosis of Infectious Diseases: A Multicenter Retrospective Cohort Study. Clin Infect Dis. 2021;72(2):239-245. doi:10.1093/cid/ciaa035

**Results:**

During the study period 123 tests were ordered (106 at IH and 17 at UU) (Table 1). Most were obtained via ID consult (118/122). Testing incidence at IH was 3.24/100 ID consults and at UU was 0.3/100 ID consults. At least one organism was identified in 44% (47/106) of tests at IH and 70% (12/17) at UU (p=0.06). Median TAT was 2 days at IH vs 8 days at UU (p < 0.05, Figure 1). Clinical impact varied by criterion between institutions (Table 2). Overall, mcfDNA testing had no discernible clinical impact in 64% (68/106) of IH cases and 76% (13/17) UU cases, (p= 0.41). A positive change in management resulted after 14% (15/106) of tests at IH and 23% (4/17) of tests at UU, p=0.46. Ordering and clinical impact varied by clinical indication (Table 3).

Tests by clinical indication

**Figure:**
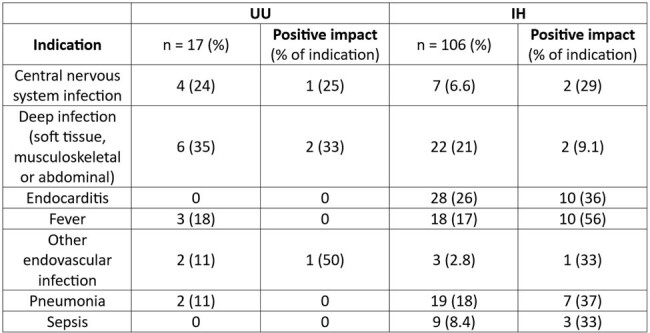

UU, University of Utah; IH, Intermountain Health

**Conclusion:**

At UU, test ordering was more selective but yielded higher test positivity. At IH, where ordering practices were slightly more liberal, a clinical diagnosis was more often confirmed by mcfDNA results. Despite differences in testing availability and strategy, positive impact percentages were similar between institutions. At IH, mcfDNA results positively changed management for more patients, at the cost of more tests sent overall. Further research is needed to further clarify best mcfDNA testing strategies.

Turnaround time

**Figure:**
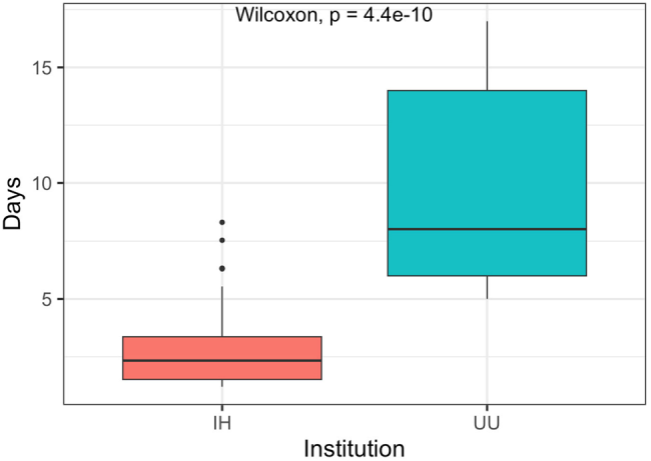

UU, University of Utah; IH, Intermountain Health

**Disclosures:**

Bert K. Lopansri, MD, D(ABMM), FIDSA, Cepheid: Grant/Research Support|Seegene: Advisor/Consultant

